# Advancing biomedical science through investments in elite training

**DOI:** 10.1371/journal.pone.0272230

**Published:** 2023-02-02

**Authors:** Misty L. Heggeness, Donna K. Ginther, Maria I. Larenas, Frances D. Carter-Johnson

**Affiliations:** 1 U.S. Census Bureau, Washington, DC, United States of America; 2 School of Public Affairs & Administration, University of Kansas, Lawrence, Kansas, United States of America; 3 Department of Economics, the University of Kansas, Lawrence, Kansas, United States of America; 4 Inter-American Development Bank, Washington, DC, United States of America; 5 National Science Foundation, Alexandria, Virginia, United States of America; University of Naples L’Orientale, ITALY

## Abstract

How can governments invest in the public good of science in a way that accelerates advancement and encourages innovation at the frontier of science–all the while acknowledging that investing in science means investing in scientists? The Ruth L. Kirschstein National Research Service Award (NRSA) program is a research-training program administered by the National Institutes of Health (NIH) that makes such investments. This study examines the impact of NRSA postdoctoral fellowships on subsequent career outcomes using NIH administrative records on applicants for the fellowship from 1996 to 2008. It finds that fellowships increased the probability of receiving subsequent research awards from 4.0 to 6.3 percentage points and of achieving a major independent research award from 2.6 to 4.6 percentage points. The findings demonstrate that federally funded fellowships promote the retention of scientists in the biomedical research workforce.

## Introduction

Governmental agencies have advanced the frontier of biomedical research and innovation since the early 1900s –acknowledging both the value and need to support scientific advancements for the betterment of humankind. Scientific advancements are a public good. Scientific funding is risky. Left to the private sector, funders would lean towards more risk-averse projects that are more likely to guarantee publications and profits but not necessarily transformative scientific discoveries. Most scientific funding agencies like the National Institutes of Health (NIH), the largest funder of biomedical research, have a mission that explicitly acknowledges the importance of funding and training the next generation of scientists. However, a fundamental question remains: How effective is it to invest in young scientists through fellowships and other training grants? Do the returns outweigh or at least equal the investment?

While measuring the actual returns is a monumental challenge, it is possible to measure a related question by examining whether early investments in biomedical training keep scientists engaged in the pursuit of future NIH funding to advance new knowledge and *leaning* into leading federally funded biomedical research grants in a way they otherwise would not. Others have studied the dynamics of transition from graduate training to research independence by becoming a principal investigator [1–3]. In this paper, we use the NIH’s Ruth L. Kirschstein National Research Service Award (NRSA) to study the effect of an elite fellowship program on subsequent career outcomes. We ask whether those who received the award were more likely to stay engaged in federally funded biomedical science research later in their career, which we define as the probability of applying for future NIH grants and becoming a principal investigator on an R01-equivalent grant.

The NIH is the primary source of U.S. federal funding for the biomedical research system. It awards upwards of $30 billion annually in grants to academic institutions across the country for the advancement of basic science research, translational research, and related endeavors. While the NIH has a robust grant award system funding a range of activities from predoctoral studies to major grants of Nobel prize winners, its most prestigious grant awards defining independence are the R01 and equivalent grants. These awards fund salaries of principal investigators, scientific research staff, postdocs, and graduate students. They also fund equipment, lab creation and development, and other expenses related to the science like travel for dissemination of key findings or support for the publication process. Grants are funded for no more than five years with renewal eligibility.

Recent studies have examined the impact of funding on research careers [4–9]. These studies from across the globe describe environments where the type of research funding, duration of training, and mentor support during graduate school is correlated with performance and independence later in one’s scientific career. While these studies advance the general knowledge of the relationship between early training and successful career independence, they are limited either in sample size (and, therefore, generalizability) or complete data along the path of independence to understand the mechanisms through which careers rise. For example, one study examines early career physician-scientists using a sample of pediatric urology and linking graduates from 1985 to 2016 to grant receipt using the NIH Research Portfolio Online Reporting Tool [7]. While this is valuable in assessing how many trained pediatric urologists received future grants, the lack of data regarding who applied for funding creates a gap in our understanding of the pathway that we attempt to fill with our research.

Our paper contributes two novel advancements in this field of study. The first is a technical advancement. The de jure method of awarding funding is consistent with a regression discontinuity design (RDD) [10–12]. RDD methods are appropriate when there is an arbitrary or exogenous rule, usually some type of score, within a program, delineating who gets in and who does not. In this case, one can effectively evaluate outcomes for those who just got in (whose score was just high enough) relative to those who just missed getting in (whose score was just below the cutoff). However, just as researchers need to test for pre-trends in difference-in-difference (DID) models, they also must examine RDD assumptions. We found that de jure is not always de facto, and prior to estimating an RDD it is important for researchers to test the assumptions. If sharp or fuzzy RDD assumptions are violated in funding mechanisms, then other methods should be used. We discover that for federally funded training programs that use peer review and criterion scores, using a regression discontinuity design to study the impact of federal funding is not appropriate, mostly because of the two-stage process for assigning awards. Second, we find that federally funded elite fellowships are a worthwhile investment for keeping young scientists engaged in scientific research careers. The experience young scientists have applying for and participating in the independent research fellowship process imprints a valuable experience that helps them acquire more independent NIH research grants in the future.

### Case study of an elite fellowship program

Since the mid-1970s, the National Institutes of Health (NIH) has formally committed to training high-potential, early -career scientists to carry out the nation’s biomedical research agenda through the congressionally mandated Ruth L. Kirschstein National Research Service Award (NRSA). While the program is subject to periodic review [13], few researchers have specifically evaluated the impact of these types of advanced training investments on subsequent career success in acquiring research funding and advancing science [14–17].

This study uses data from this elite fellowship program to provide evidence towards two important questions. *First*, *does early career advanced training affect the likelihood of future NIH funding*? This is measured by whether a fellowship applicant or awardee applied for or received a research grant award from NIH, the number of awards, and whether they appear as an award applicant four years or more from the time they first applied for the fellowship program. *Second*, *does a highly competitive fellowship award contribute to an independent federally funded research career*? We recognize that an independent research career can mean many things to many people. And while there do exist very successful independent researchers in innovation hubs like Howard Hughes Medical Institute (HHMI) within the U.S., for the purposes of this paper we define an independent federally funded research career of individuals who have applied for and/or received an NIH R01 or equivalent grant. NIH R01 and equivalent grants are the most prestigious NIH grant available for scientific researchers who lead research and investigations on the cutting-edge of science. NIH is, after all, the largest U.S. funder of biomedical research and, as such, holds the largest market share on supporting and advancing independent research careers in the U.S. We measure this outcome by estimating the probability of receiving a major independence milestone award for biomedical researchers, the NIH R01 award, four years or more after applying to the fellowship program [18, 19]. Using the scoring of fellowship applications and matching techniques, we analyze the outcomes of individuals who received the fellowship award and those whose application scores and observable characteristics are similar, but who did not receive an award.

If those receiving the fellowship are more likely to remain in science and have an independent research career, then this is evidence that federal governments can effectively invest in the public good of science through supporting early career training. Programs similar to the NRSA promote and encourage research independence while at the same time training young scientists to write grants. This imprints on them the relevant techniques and knowledge for a future that includes successfully funded research projects. By encouraging young scientists to *lean in* early through independently applying for training funding, the federal government can continue to advance the scientific frontier.

### Background

#### The evolution of government support for early career scientific training

The National Institutes of Health (NIH) NRSA program has multiple award mechanisms that fund research training in biomedical science [20–22]. These include institutional training programs (T-series awards) for undergraduates, graduate students, and postdocs, as well as individual fellowships (F-series awards) for graduate students pursuing a PhD or postdoc positions. Since its inception in 1974, these fellowships have been highly competitive. In 2008, the federal government allocated around $751.2 million in funding to directly support biomedical research training for 16,370 undergraduate students, graduate students and postdoctoral researchers via T-series and F-series awards at NIH [23]. Of these awardees or appointees, 1,487 (around 9.1 percent) received an NRSA F32 postdoctoral fellowship award. Our analysis focuses on the most elite competitive award, the F32 postdoctoral fellowship, which provides funding for the transition toward research independence. This award signals to institutions that the early career scientist is independently able to secure funding of unique research ideas. Anecdotally, young scientists claim that high-ranking institutions prefer early career scientists who obtain external signals like elite awards in early career.

**Fig 1:**
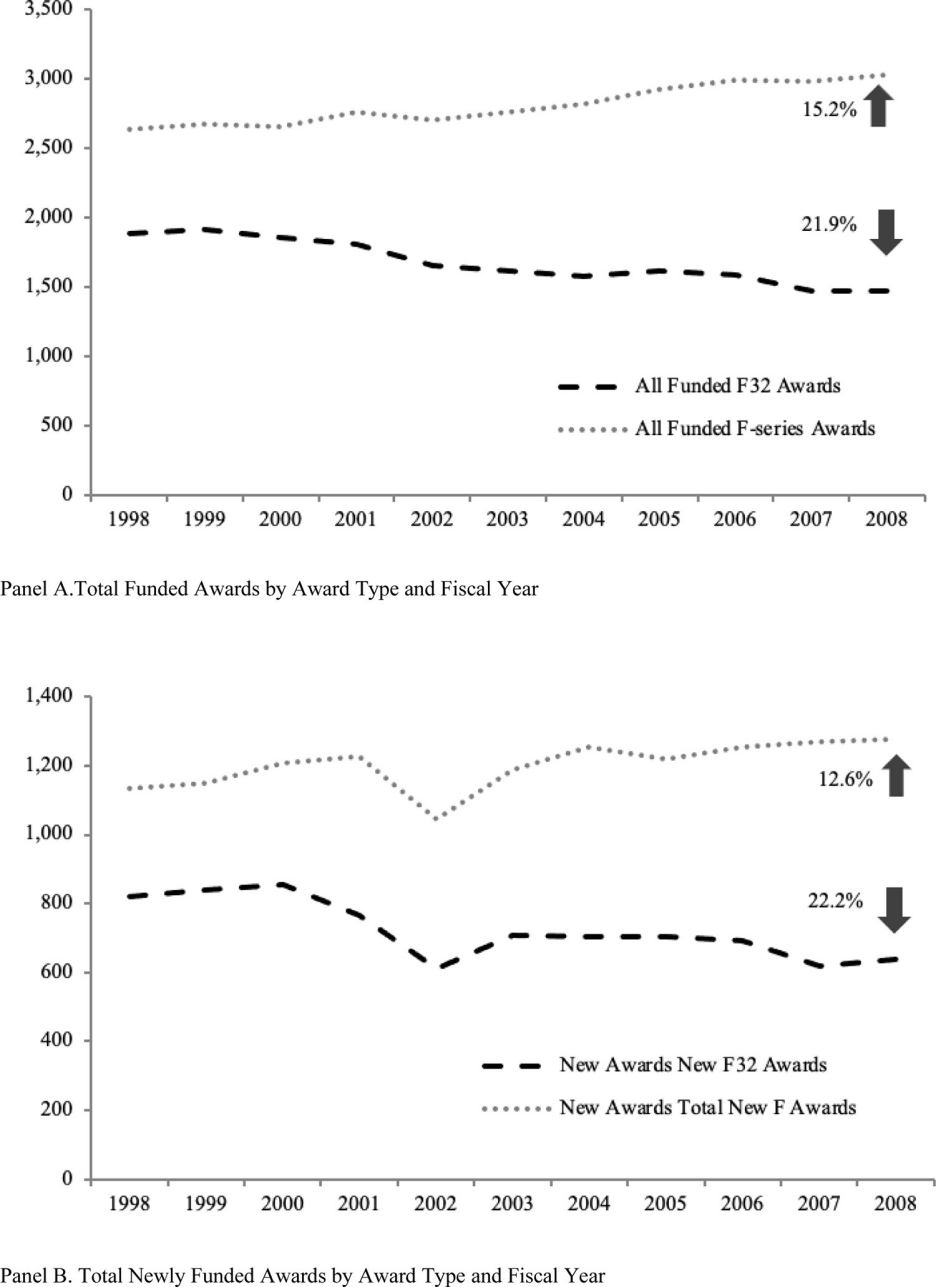
Total NRSA F-series awards and F32 awards funded by NIH by year, 1998–2008. (A) Awards funded by award type and fiscal year regardless of competitive status. (B) Total newly funded awards by award type and fiscal year [24]. Source: NIH IMPAC II, National Science Foundation Doctoral Record File.

Between 1998 and 2008, the number of F-series fellowship awards funded increased by 15.2 percent, as shown in Panel A of Fig 1. While the total number of F-series awards increased, the F-mechanism diversified by funding more positions in other F-series awards, causing the total number of elite NRSA F32 postdoctoral fellowship awards funded in any given year to decrease over the period by 21.9 percent (Fig 1, Panel A).

Even though overall the F-series fellowship awards have experienced growth over the period of our study, they grew much more slowly than the number of PhDs conferred in biological sciences during that time. According to the National Science Foundation, the number of new PhDs in biomedical sciences grew annually from 5,838 in 2000 to 7,797 in 2008, an increase of 33.6 percent [25]. This rapid growth in PhD scientists is a byproduct of the doubling of federal funding for biomedical research at the turn of the 21^st^ century. The competing needs of the scientific enterprise to increase cheap labor (students and postdocs) to advance science are a conundrum in scientific circles [26]. While competition to receive an award has increased with the number of new biomedical PhDs, it has also increased pressure on funding agencies to understand the value of these programs.

#### The effect of postdoctoral study on career outcomes

There are a limited number of studies that have examined the impact of a postdoctoral appointment (postdoc) on later career outcomes. Those that do combine academic fields, which makes it difficult to generalize the results to particular fields of science [27–30]. These limited studies have investigated the impact of a postdoc on subsequent placement in academic careers. Longer duration postdocs are not associated with improved academic placements [27]. One study examined the association between earlier cohorts of federally funded postdocs and their subsequent biomedical careers and found significant gender differences in outcomes; women were more likely to leave scientific research careers than men [28]. Career trajectories of women appeared to be affected by measures related to their mentor’s quality. For female postdocs, the higher the mentor’s h-index, the more likely the postdoc was to receive an NIH grant. That study does note the possibility that results may not be extrapolated to more recent NRSA cohorts [28].

Some studies, however, indicate a negative impact of postdoctoral and graduate appointments. One study found that individuals who took a postdoc in biomedical fields had lower lifetime earnings than those who skipped the postdoc [31]. Although the postdoc was useful for obtaining an academic tenure-track research position, the likelihood of achieving this goal dropped considerably over time. Another study used the Survey of Earned Doctorates to examine whether graduate research assistantships were more effective at launching research careers than graduate student support by federally funded biomedical training grants or fellowships [32]. Graduate students with research assistantships had more successful research outcomes in terms of research-focused jobs although this study did not address postdocs funded by different mechanisms.

The evidence on the impact of postdoctoral and graduate training on later career outcomes in general is mixed, and the evidence on the impact of federally funded biomedical science postdoctoral fellowships using an adequate methodology is limited, prompting this study. That said, studies assessing the impact of federal investments in training the biomedical research workforce, including evaluation studies commissioned by the federal government, show that individuals participating in training programs experience higher rates of remaining in scientific research in comparison to their counterparts who did not receive formal training [14, 15].

This study is most closely related to studies on the impact of NIH postdoctoral training [16] and research grant funding [17] on scientific productivity. A study using a regression discontinuity showed that receiving a postdoctoral fellowship increased publications by 20 percent in the next five years relative to non-awardees [16]. In addition, postdoctoral fellows received higher dollar amounts in subsequent funding compared to non-awardees. Our analysis below indicates that RDD has limitations for estimating the impact of the postdoctoral fellowship awards. A detailed discussion of the application, review and award process discussed below delineates our critique of the RDD as a valid method for analysis of the F32 fellowship.

#### The independent researcher postdoctoral proposal process

A thorough understanding of the NRSA application and award process is necessary for the development of our empirical approach. The goal of the award is “to enhance the research training of promising postdoctoral applicants who have the potential to become productive, independent investigators in scientific health-related fields…” In defining a postdoctoral position for the purposes of this paper, we follow the guidance of federal agencies who, in 2007, defined a postdoc as “…an individual who has received a doctoral degree (or equivalent) and is engaged in a temporary and defined period of mentored, advanced training to enhance the professional skills and research independence needed to pursue his or her chosen career path” [33]. One mechanism for accomplishing this goal is through funding a postdoctoral fellowship position in a mentor’s scientific laboratory. Fellowships are allocated based in part on a score assigned during peer review, which considers the merit of an applicant’s research proposal and the quality of proposed training and career development support available through the institution and mentors. In addition, before offering an award, NIH program staff perform a thorough review to ensure the validity of the research idea and the quality of the training environment. Staff also verify whether the research proposed matches well with the NIH institute’s scientific needs and priorities.

**Fig 2:**
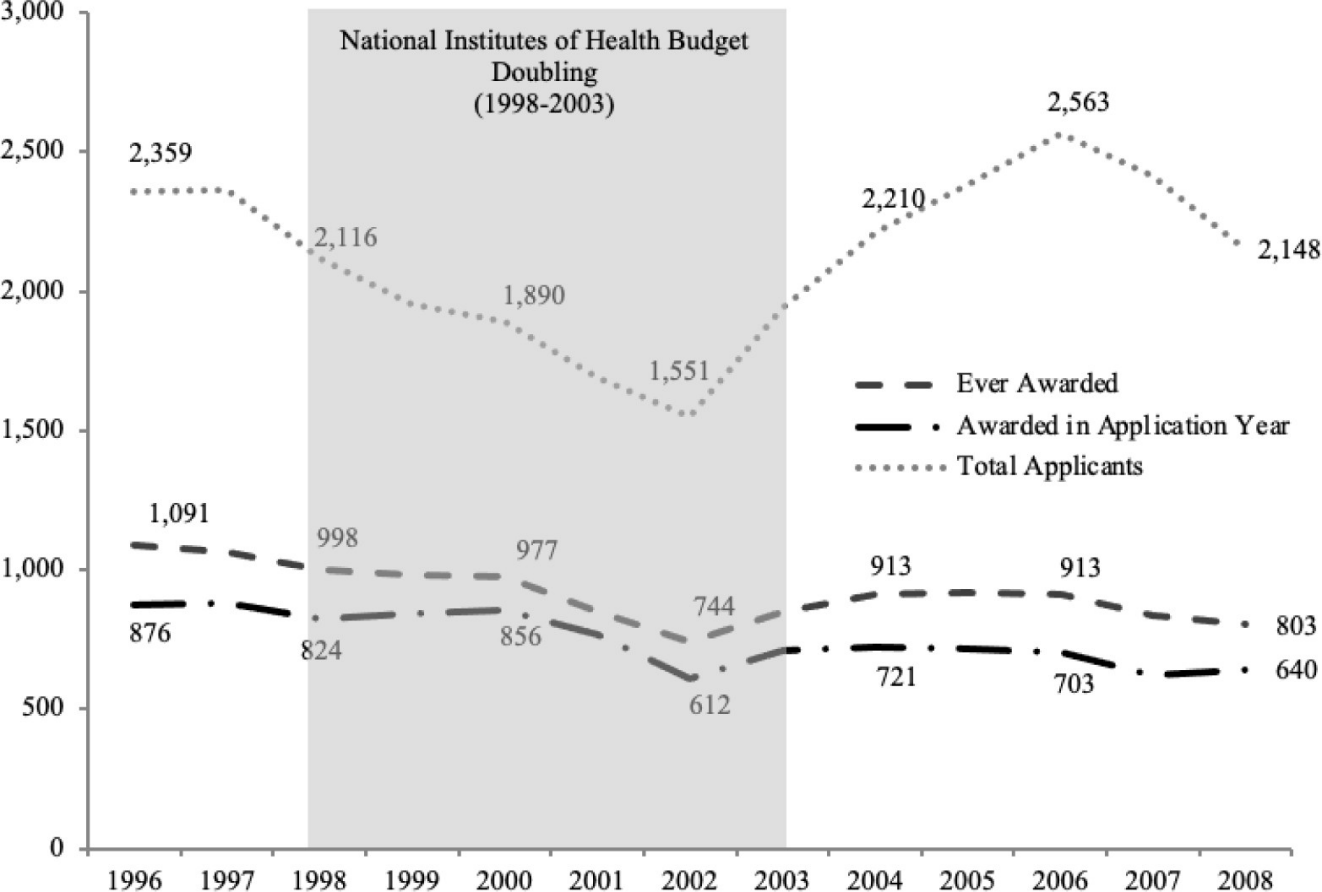
Total new NRSA F32 applicants, individuals ever awarded, and individuals awarded in same application year. Data shown for fiscal year of application, 1996–2008. Source: Author calculations based on NIH IMPACII administrative records [35].

Fig 2 shows the number of applications by year. The number of new applications ranged from around 1,500 to 2,500 per year in the mid-2000s. During the NIH Doubling (1998–2003), [34] the number of new applications decreased from around 2,100 in 1998 to a low of approximately 1,500 in 2002. This may reflect the increase in the budget during the doubling that likely increased postdoctoral slots on funded research grants. After the doubling, the number of new applicants once again began to rise and peaked in 2006. In 2008, one award cost, on average, around $48,998.

The authors posit that applicants who meet the basic requirements for the award are willing to apply if the benefit of winning is higher than the cost of applying. Applicants are required to have a research doctorate or professional degree by the time of the award, be a citizen or a permanent resident of the United States and have a sponsor (who functions as a mentor), and a sponsoring institution. All these requirements limit the size of the applicant group and make it more homogeneous, limiting the impact of unobserved characteristics on the probability of receiving an award.

Selection to receive a fellowship award depends on multiple measures. First, a scientific review group evaluates each new application for scientific merit. At the NIH, for the most part, the Center for Scientific Review (CSR) develops and runs the group. CSR is an independent body that provides institutes services related to peer review, as well as documenting the evaluation and scoring of applicants. The scientific review groups include accomplished scientists and experts as external reviewers. The evaluation considers observable characteristics of the applicants, *A* (the applicant’s past academic and research records, publications); observable characteristics of the sponsor, *S* (the sponsor’s general qualifications); research potential, *Pot* (references, the applicant’s research goals); research environment, *E* (training environment); the research proposal, *P*, plus the perception of the reviewer in that period *ε*_*qt*_. Each reviewer, *r*, assigned to an application, *i*, for institute or center, *j*, in a particular year, *t*, and council round, *q*, gives a preliminary reviewer overall review score, *PS*_*ijrtq*_, for each application defined as:

$${PS}_{ijrtq}=f(A,S,Pot,E,P,\varepsilon_{rtq})$$ (1)

$${PS}_{ijtq}=\frac{\sum_{n=1}^{r}{PS}_{ijrtq}}{\sum_{n=1}^{r}N_{ijrtq}}$$ (2)

Eq 2 gives a final overall review score, *PS*_*ijtq*_, for each discussed application, *i*, who applied to an institute or center, *j*, in year, *t*, in council round, *q*, and is simply determined by the mean score of all scientific review members’ preliminary scores. Review panels score proposals from best to worst, where the lowest scores are considered the best. Until 2009, proposal review scores ranged from 100 (high impact) and 500 (low impact). Starting in 2009, a process called Enhancing Peer Review changed the scale of evaluation to 10–90 where the final overall impact scores now range from 10 (high impact) through 90 (low impact) [36].

Prospective awardees are evaluated in council round, *q*, in a calendar year, *t*. At the end of the scoring process, the scientific review group reports the review scores to the institute or center’s selection process. At the end of the first selection, those applications with the lowest scores (the best applications) receive a secondary level of review by staff. This step is a key determinant of the funding decision. Program staff examines the applications’ summary statements and review scores and weighs these factors against the needs and priorities of the institute or center. The director (or their delegate) makes the final decision as to whether to offer an award.

Applicants who do not receive an award typically explore multiple options, which include appointment to a position as postdoctoral researcher on a mentor’s research grant, appointment to a training grant if a slot is available, application to other funding sources such as foundation grants, or, if feasible, resubmission of their application. Other scenarios include leaving academic research or exploring other career paths. The true value of elite fellowship awards is their power in developing a skillset to lead and conduct a research project and the freedom it gives the postdoc to engage in independent research under the supervision of their mentor. We are testing whether funding to develop and pursue a high-quality research project early in one’s career has an effect on the probability of receiving future biomedical research funding and establishing an independent research career.

A sharp regression discontinuity design is appropriate if the majority of proposals are funded in the order of the score. Aberrations in the funding of proposals with review scores near the cutoff constitute a fuzzy regression discontinuity design. However, if institutes skip several (competitive) low-scoring proposals and reach for higher scoring proposals that are more consistent with institute priorities or because of other administrative hurdles on low-scoring applications, then an RDD has limitations. This paper now turns to the data before evaluating the appropriate methods for determining the impact of the F32 award on subsequent career outcomes.

## Materials and methods

### Data

The authors use NIH administrative records from 1996 to 2008. NIH matches its administrative records to data from the National Science Foundation’s (NSF) Survey of Earned Doctorates (SED), an annual census of doctoral recipients from U.S. institutions [37]. The NSF SED contains information on individual demographics, characteristics of graduate study, and future career plans. By linking these datasets, missing data and additional individual-level covariates are added to the sample. The authors used demographic variables before or at the point of PhD completion including age at PhD completion, gender, race/ethnicity, marital status at PhD completion, PhD field of study, and type of doctorate education funding. The two data sources were used to construct one large panel dataset for analysis, limiting the sample to those who applied for NRSA F32 funding between 1996 and 2008, and then observing these individuals’ application and funding patterns through 2015. These data are from the same sample used in another study [38]. University of study and publications pre-terminal degree are viewed as relevant factors when considering funding decisions [39]. In our study, we use the review score of the grant application in our analysis, which contains valuable information about prestige of graduate institute and early publications since reviewers use that information to evaluate and score the grant. Individuals from more prestigious universities and with early publications score higher on grant applications, on average. We also assume that PhD supervisor reputation and quality are captured, at least indirectly, in the review score of future grants.

This analysis uses detailed fellowship and subsequent grant application and funding information, including application review score, funded or non-funded status, timeframe and institute receiving applications or funding the award, as well as previous grant funding or training affiliations. We include outcome variables like NIH’s Research Project Grants (RPGs) and R01-equivalent grants. RPGs are a broad classification of multiple award mechanisms, including the R01-equivalent. R01-equivalent is considered the most prestigious award demonstrating independence and leadership in science. This information on NRSA F32 postdoctoral fellowship applicants from 1996 to 2008 comes from NIH administrative records [35]. The authors further queried administrative records for subsequent applications for funding and awards for these individuals. As in a similar study [16], this paper defines the outcome variables to identify research award application or receipt four or more years out from the individual’s NRSA F32 application year.

Table 1 shows descriptive statistics for all fellowship applications from 1996 to 2008 by award status. Awardees and non-awardees differ across a number of observable characteristics. Awardees are younger, more likely to be married and white. Awardees are significantly less likely to be Black or Hispanic. Individuals with MD degrees are less likely to receive fellowship awards whereas PhDs are more likely. Individuals with biomedical or social science degrees are more likely to receive fellowship awards compared with those whose PhD field is not reported. Individuals who have had predoctoral traineeships are more likely to receive awards. Awardees are significantly more likely to aspire to and to receive subsequent NIH funding as measured by the number of applications and awards, the probability of a research program grant award, and the probability of an elite independent research grant award. As expected, awardees have significantly lower (better) scores on their last observed F32 application.

**Table 1:** Descriptive statistics of all applicants by funding status, 1996–2008.

		All	F32 awarded	No F32 awarded	t-test	p-value
	Review score	220.67	175.81	258.38	104.64	0.000
		(75.291)	(44.581)	(75.162)		
DEMOGRAPHICS						
	Age at application	31.201	30.989	31.363	4.04	0.000
		(7.629)	(6.776)	(8.218)		
	Age at application missing	0.042	0.033	0.048	6.00	0.000
		(0.200)	(0.180)	(0.214)		
	Married at application	0.381	0.394	0.371	-3.97	0.000
		(0.486)	(0.489)	(0.483)		
	Married at application missing	0.205	0.181	0.223	8.62	0.000
		(0.404)	(0.385)	(0.416)		
	Female	0.417	0.417	0.416	-0.19	0.850
		(0.493)	(0.493)	(0.493)		
	Sex missing	0.051	0.037	0.061	9.18	0.000
		(0.219)	(0.188)	(0.240)		
	White, non-Hispanic	0.344	0.384	0.314	-12.17	0.000
		(0.475)	(0.486)	(0.464)		
	Black, non-Hispanic	0.009	0.006	0.011	4.60	0.000
		(0.095)	(0.077)	(0.106)		
	Asian, non-Hispanic	0.086	0.084	0.087	0.97	0.330
		(0.280)	(0.277)	(0.282)		
	Other, non-Hispanic	0.002	0.003	0.002	-1.97	0.049
		(0.048)	(0.054)	(0.042)		
	Hispanic	0.032	0.029	0.034	2.46	0.014
		(0.175)	(0.167)	(0.181)		
	Race missing	0.544	0.512	0.569	9.40	0.000
		(0.498)	(0.500)	(0.495)		
EDUCATION and TRAINING						
	MD	0.086	0.080	0.090	3.09	0.002
		(0.280)	(0.271)	(0.286)		
	MD/PhD	0.032	0.034	0.031	-1.50	0.134
		(0.176)	(0.181)	(0.172)		
	PhD	0.867	0.876	0.860	-3.95	0.000
		(0.340)	(0.330)	(0.347)		
	Other Degree	0.016	0.011	0.020	5.93	0.000
		(0.125)	(0.103)	(0.139)		
	Biomedical degree	0.594	0.619	0.575	-7.40	0.000
		(0.491)	(0.486)	(0.494)		
	Physical Science degree	0.129	0.124	0.133	2.20	0.028
		(0.335)	(0.329)	(0.339)		
	Social Science degree	0.069	0.074	0.065	-2.69	0.007
		(0.253)	(0.261)	(0.247)		
	Prior T32 Predoc appointment	0.021	0.025	0.018	-3.74	0.000
		(0.144)	(0.156)	(0.134)		
	Prior T32 Postdoc appointment	0.019	0.016	0.021	2.76	0.006
		(0.136)	(0.127)	(0.143)		
	Prior NRSA Predoctoral Fellowship	0.001	0.001	0.000	-0.51	0.612
		(0.023)	(0.024)	(0.021)		
OUTCOME VARIABLES						
	Number of RPG Awards	0.387	0.555	0.259	-22.95	0.000
		(1.071)	(1.243)	(0.894)		
	Number of RPG Applications	1.940	2.664	1.388	-24.32	0.000
		(4.36)	(5.03)	(3.68)		
	Probability of RPG	0.183	0.256	0.126	-28.01	0.000
		(0.386)	(0.437)	(0.332)		
	Probability of R01	0.133	0.192	0.088	-25.56	0.000
		(0.339)	(0.394)	(0.283)		
	Probability of Never Receiving an RPG	0.678	0.581	0.752	30.59	0.000
		(0.467)	(0.493)	(0.432)		
N		27,580	11,938	15,642		

*Source*: Authors’ calculations. National Institutes of Health IMPACII and NIH/NSF Survey of Earned Doctorates, 1996–2008.

The analysis sample is a subset of the full sample. All applications that are higher than the 60th percentile in each council round are dropped because scores for these applications are not consistently saved in the reporting database and practically none of them get funding. Some institutes and centers have too few applicants for our preferred analysis method, so we drop applicants from seven institutes and centers for this reason. Too few applicants generally implies that there were fewer applicants than available funding and, as such, all individuals who applied received funding or the applicant pool was fewer than 10 applications. Council rounds prior to fiscal year 1996 are incomplete and dropped from the analysis sample. The final analysis sample contains 14,276 individuals, and descriptive statistics are reported in Table 2. In the analysis sample, awardees and non-awardees no longer differ in terms of age at application, marital status, or likelihood of having a prior traineeship. In the next section, this paper examines whether institutes fund proposals in the order of the score and whether a regression discontinuity design (RDD) is warranted.

**Table 2:** Descriptive statistics of applicants by funding status, analysis sample, 1996–2008.

		All	F32 awarded	No F32 awarded	t-test	p-value
	Review score	220.67	162.244	193.68	60.22	0.000
		(75.291)	(26.899)	(34.432)		
DEMOGRAPHICS						
	Age at application	31.201	30.942	31.078	1.12	0.261
		(7.629)	(6.775)	(7.115)		
	Age at application missing	0.042	0.034	0.037	0.98	0.328
		(0.200)	(0.181)	(0.189)		
	Married at application	0.381	0.396	0.38	-1.84	0.066
		(0.486)	(0.489)	(0.485)		
	Married at application missing	0.205	0.182	0.186	0.68	0.494
		(0.404)	(0.386)	(0.389)		
	Female	0.417	0.412	0.424	1.30	0.194
		(0.493)	(0.492)	(0.494)		
	Sex missing	0.051	0.038	0.055	4.90	0.000
		(0.219)	(0.190)	(0.228)		
	White, non-Hispanic	0.344	0.386	0.319	-7.93	0.000
		(0.475)	(0.487)	(0.466)		
	Black, non-Hispanic	0.009	0.006	0.01	2.64	0.008
		(0.095)	(0.075)	(0.098)		
	Asian, non-Hispanic	0.086	0.085	0.079	-1.29	0.196
		(0.280)	(0.279)	(0.269)		
	Other, non-Hispanic	0.002	0.002	0.002	-0.33	0.744
		(0.048)	(0.050)	(0.047)		
	Hispanic	0.032	0.029	0.029	-0.07	0.946
		(0.175)	(0.168)	(0.167)		
	Race missing	0.544	0.509	0.579	7.97	0.000
		(0.498)	(0.500)	(0.494)		
EDUCATION and TRAINING						
	MD	0.086	0.084	0.08	-0.74	0.459
		(0.280)	(0.277)	(0.272)		
	MD/PhD	0.032	0.035	0.03	-1.80	0.073
		(0.176)	(0.185)	(0.170)		
	PhD	0.867	0.87	0.874	0.67	0.506
		(0.340)	(0.336)	(0.332)		
	Other Degree	0.016	0.01	0.016	2.76	0.006
		(0.125)	(0.102)	(0.125)		
	Biomedical degree	0.594	0.614	0.617	0.33	0.739
		(0.491)	(0.487)	(0.486)		
	Physical Science degree	0.129	0.129	0.124	-0.72	0.471
		(0.335)	(0.335)	(0.330)		
	Social Science degree	0.069	0.074	0.071	-0.67	0.506
		(0.253)	(0.261)	(0.256)		
	Prior T32 Predoc Award	0.021	0.024	0.027	0.96	0.339
		(0.144)	(0.154)	(0.162)		
	Prior T32 Postdoc Award	0.019	0.016	0.02	1.65	0.099
		(0.136)	(0.127)	(0.141)		
	Prior NRSA Predoctoral Fellowship	0.001	0.001	0	-1.64	0.101
		(0.023)	(0.023)	0.000		
OUTCOME VARIABLES						
	Number of RPG Awards	0.387	0.586	0.367	-10.24	0.000
		(1.071)	(1.281)	(1.095)		
	Number of RPG Applications	1.94	2.752	1.698	-12.51	0.000
		(4.360)	(5.158)	(4.066)		
	Probability of RPG	0.183	0.266	0.165	-13.69	0.000
		(0.386)	(0.442)	(0.372)		
	Probability of R01	0.133	0.204	0.122	-12.25	0.000
		(0.339)	(0.403)	(0.328)		
	Probability of Never Receiving an RPG	0.678	0.579	0.713	16.02	0.000
		(0.467)	(0.494)	(0.452)		
N		27,580	9,276	5,000		

*Source*: Authors’ calculations. National Institutes of Health IMPACII and NIH/NSF Survey of Earned Doctorates.

### Methods

#### An analysis of awarding behavior

Each institute has discretion in deciding who is offered a fellowship based on the current research priorities of the institute. Institutes and centers vary in their process for awarding a fellowship. For this study, the authors interviewed staff at various institutes and centers who provided a representative view of the variation in procedures across the organization. Through interviews, the authors learned that the process for awarding fellowships is more complex than funding proposals with the best scores.

The review and scoring of fellowship proposals and institute budget realities create scoring thresholds that theoretically would support the use of a regression discontinuity design (RDD) as in a similar study [16]. RDD is appropriate when the forcing mechanism, in this case the review score, is the principal factor determining who gets and does not get funding. The score or budget line is determined based on the availability of funds. In such cases, one can evaluate units around the cut off variable (e.g., budget line) to identify the impact of the program for those who just got in compared to those who just did not. Decisions of who to fund are made at the year-institute-council round level. We demonstrate that using only year and institute to estimate an RDD model as has been previously done [16] is not an optimal method for evaluating training fellowships given the fellowship selection process.

If awards are based solely on the review score allocated from best (lowest) score to worst (highest) score until the institute budget allocations for the fellowship are exhausted, the percent of applicants selected out of order would be zero or close to zero. If this were the case, a sharp RDD would be valid because, in this scenario, the institute follows the guidance of the peer review process for the best (lowest) scores. The pay line determines where all resources have been spent within the institute. Suppose there are 10 applicants arranged in order from 1 (the best) to 10 (the worst) proposal scores. If the budget allows for funding 4 applicants, the pay line is 4 and anyone with a score of 4 or less would be funded. If, however, an institute has funded a majority of applicants with meritorious scores and some discretion is used regarding awards to applications with scores near the pay line, we would observe minimal disorder in funding. In this example, the institute uses discretion to potentially skip some applicants close to the pay line in order to fund applications with slightly worse scores but with a better fit within their scientific priorities and where the institute staff believes the applicant has the best-case scenario for future success. If this were the case, a fuzzy RDD would be appropriate. Alternatively, institutes could just choose to use a significant amount of discretion when selecting proposals for funding in order to meet institutional goals related to scientific priorities or, perhaps, diversity of the workforce. For institutes that engage in this behavior, no real cutoff exists and an RDD is not appropriate. Our prior findings [38] demonstrate both visually and through data analysis that overall discretion in NIH funding of independent research awards overrides decisions made by a cutoff score sufficiently that neither a sharp nor fuzzy RDD model cannot be used as a valid method.

We interviewed program officers from four institutions ranging in topic, size, budget, and programmatic content. The interviews lasted around one hour each and were focused on identifying the administrative steps that lead to funding decisions starting from grantee submissions to review (which is where grants get discussed), scoring, rules for complete applications, budget office interactions with program staff, and final award decision making. Interviews with NIH staff indicated that the funding process is multifaceted. The institute receives an application’s peer review score, which, in most cases, is defined by the study section through coordinated scientific review groups. Once the institute receives the scores, staff assesses the full application, including the summary statement from peer review, the quality of the training institution, and the alignment of the research proposal with the institute’s research priorities. Program officers at each institute then generally participate in a team meeting in which they defend the proposals that best match their defined priorities. Together, the program officers, the training director, and other institute staff make a joint decision for recommendations to the director. Either the director or his/her delegate makes the final decision and signs off on which proposals to fund. Directors vary in terms of direct involvement in the consideration and final approval of proposals. Before a final decision is made and the candidates are informed, the budget office reviews and signs off on the final list of candidates, primarily making sure sufficient funds are available for the recommended awards.

**Fig 3:**
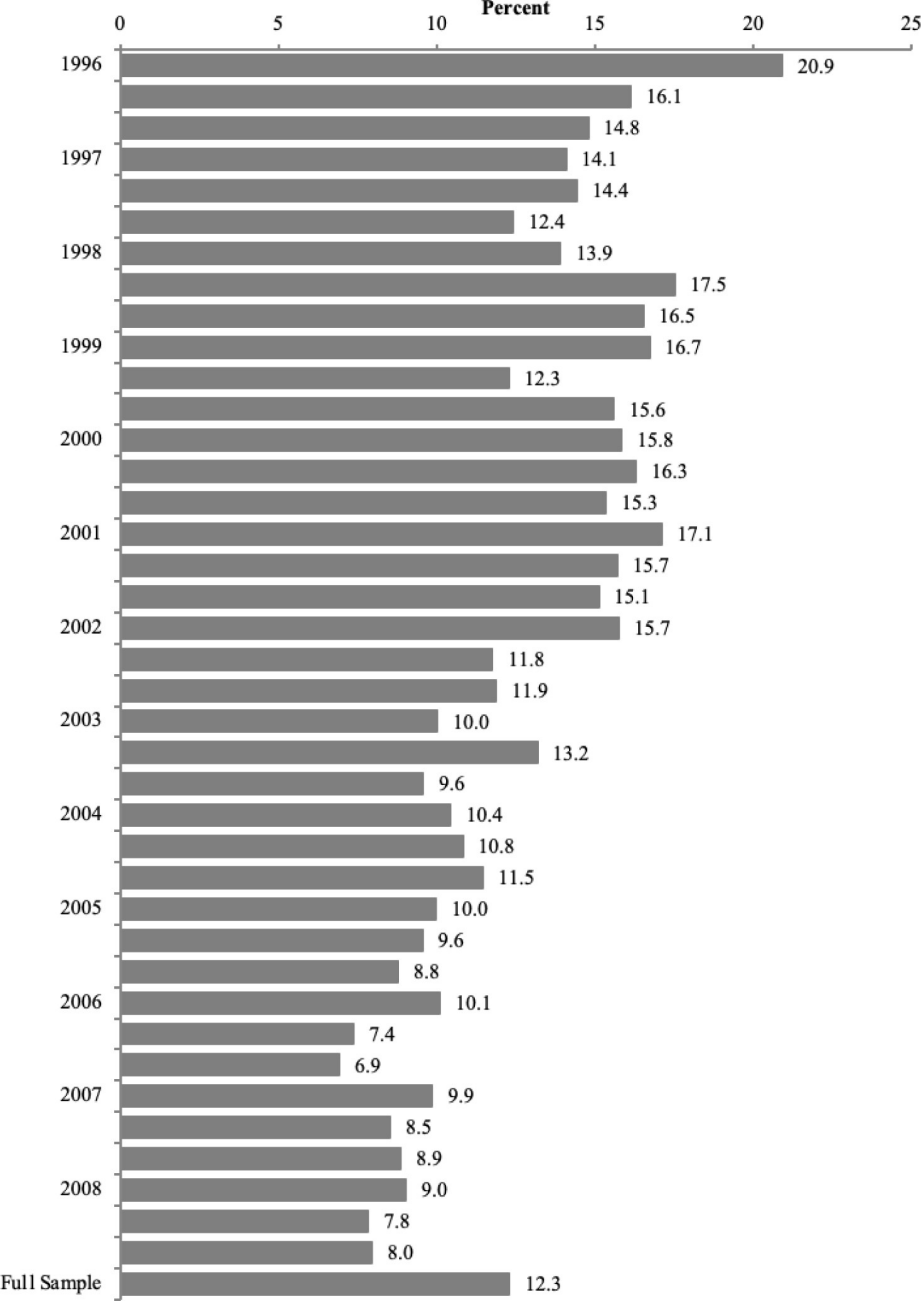
Percent of applicants selected out of order by council round, 1996–2008. Figure excludes council rounds with an N<20. Author calculations using NIH records [35].

We examined funding decisions within institutes and found a significant amount of discretion. Fig 3 illustrates why RDD is not the appropriate design for evaluating fellowship awards. It demonstrates the range of discretion used in fellowship decision-making for the full sample over the period of our study. In a given council round, anywhere from around 6.9 percent to 20.9 percent of applicants received a decision that was not based on their peer-reviewed ranked score. Either the institute funded the applicant even though other applicants had better scores, or the institute’s final decision was not to fund the applicant even though their application had a better score than others that received funding.

What does this mean for this study? Fig 3 illustrates the level of non-compliance for an RDD method and the strong tendency for discretion in decision-making within each council round. While the level of discretion has decreased in recent years, over the entire sample, around 12.3 percent (1 in 8) of all applications within a given council round received a decision that was not in line with the peer review score. Given the large proportion of discretion used within council rounds, the authors argue that RDD is an invalid method for evaluating the true impact of early career training on later career outcomes as it relates to the NRSA F32 fellowship award.

#### Matching to identify the causal effect

As described above, the process for fellowships and research grants generates a peer review score. Given the fact that the groups of individuals applying to the awards are relatively homogenous compared to the total population, Propensity Score Match (PSM) and Nearest Neighbor Match (NNM) methods are considered for this analysis. While it is understood that any unobserved characteristics that are different between funded and unfunded confound the results, the authors argue that matching is a feasible approach for the following reasons. First, they can control for unobserved differences by institute and council round. Second, selection is made at the institute level, and any unobserved differences among applicants are also not observed at the institute level and not likely contributing to the selection process. In short, there is no self-selection of fellowship award offerings. Third, individuals who apply for funding are relatively homogeneous within the institute. They have been encouraged to apply by their mentors (which means their mentors believe they have a chance of getting the award), are committed to biomedical research careers and excel at academic pursuits. Given the unobservable variation that could exist, within this select group of applicants, it is minimal.

The authors use the potential outcomes framework employed in econometric analysis to estimate the causal effect of fellowship awards on subsequent funding outcomes [40]. Let *T*_*i*_ = 1 be the treatment when an individual’s fellowship application is funded and let *T*_*i*_ = 0 if the application is not funded. In the potential outcomes framework, individuals have two potential outcomes of subsequent funding: *Y*_*i*_(1) if the individual receives the award treatment and *Y*_*i*_(0) if the individual does not receive the award. The causal effect for individual *i* of the funding award is defined as the difference in potential outcomes *Y*_*i*_(1)−*Y*_*i*_(0). That said, individual *i* is only observed when they receive the award or they do not, and we must estimate the counterfactual outcome.

Matching methods used in this study assume that treatment is independent of the outcome conditional on covariates, *T*_*i*_┴(*Y*_*i*_(0), *Y*_*i*_(1))|*X*_*i*_, the unconfoundedness assumption. Unconfoundness means that the treatment is conditionally independent of the outcome after conditioning on observable characteristics. If unconfoundedness holds, we can define the average treatment effect in terms of potential outcomes as the expected value of potential outcomes:

$$ATE=E[Y_{i}(1)-Y_{i}(0)]$$

The average treatment effect on the subsample of the treated is defined as:

$$ATT=E[Y_{i}(1)-Y_{i}(0)|T_{i}=1]$$

Two matching methods are used to identify the ATE and ATT. We employ propensity score matching (PSM) and present the PSM results in S1 Appendix The propensity score is the probability of receiving treatment conditional on observed characteristics *e*(*X*) = Pr (*T*_*i*_ = 1|*X*_*i*_ = *x*). In order to implement this method, the propensity scores for the treated and untreated in our sample must overlap such that 0<*e*(*x*)<1. The unconfoundedness assumption cannot be tested directly; thus, the authors examine whether the propensity score has a causal effect on a pseudo-outcome that was determined prior to the treatment. Unconfoundedness is not likely to be violated if the estimated effect of the treatment on the pseudo-outcome is not significant [41]. They can evaluate the overlap assumption directly by assessing the balance of the covariates in the treated and untreated groups as well as visually inspecting the overlap in the propensity scores.

Propensity score matching has been widely used in social sciences and economics [41]. However, propensity score estimates break down if the propensity score model fits to the data too well [41, 42]. This means that the authors cannot use the review score to estimate the propensity score related to fellowship funding. We therefore use the coarsened exact matching (CEM) algorithm [43] as our primary preferred method to improve the balance of the data and nearest-neighbor methods to facilitate matching on the review score. Our results presented below use nearest neighbor matching after reducing the data using the CEM algorithm.

## Results and discussion

### Results

The analysis begins by estimating the probability of receiving an NRSA F32 award using probit models; Table 3 reports the marginal effects. In addition to covariates listed in the table, all models include controls for institute and council round. In the first column, few covariates predict the likelihood of receiving an award. A handful of age dummy variables are statistically significant as well as an indicator for sex being missing in the data. Individuals who are Black, non-Hispanic are significantly less likely to receive an award. The score is included in the second column, and most observable characteristics lose statistical significance. Those with missing sex are significantly less likely to receive funding.

**Table 3:** Probit regressions on ever receiving an award, 1996–2008.

		(1)	(2)
Review score			-0.008***
			(0.000)
*Age (missing = <26)*			
	Age = 27	-0.053	0.021
		(0.036)	(0.039)
	Age = 28	-0.034	-0.028
		(0.030)	(0.036)
	Age = 29	-0.050	-0.016
		(0.029)	(0.035)
	Age = 30	-0.041	0.002
		(0.029)	(0.034)
	Age = 31	-0.035	0.024
		(0.029)	(0.033)
	Age = 32	-0.060*	-0.005
		(0.030)	(0.034)
	Age = 33	-0.045	0.027
		(0.030)	(0.033)
	Age = 34	-0.055	0.016
		(0.031)	(0.035)
	Age = 35 or 36	-0.057	0.028
		(0.031)	(0.034)
	Age = 37 or 38	-0.085*	0.014
		(0.034)	(0.036)
	Age > 38	-0.137***	-0.019
		(0.034)	(0.037)
	Married	0.016	0.018
		(0.009)	(0.010)
	Marital status missing	0.044	0.086
		(0.085)	(0.092)
	Female	-0.009	-0.006
		(0.009)	(0.009)
	Sex missing	-0.115***	-0.103***
		(0.023)	(0.026)
	Black, non-Hispanic	-0.146**	-0.103
		(0.055)	(0.055)
	Asian, non-Hispanic	-0.025	-0.016
		(0.016)	(0.017)
	Other race, non-Hispanic	0.064	0.048
		(0.074)	(0.074)
	Hispanic	0.014	0.033
		(0.025)	(0.025)
	Race missing	0.011	0.016
		(0.010)	(0.011)
MD		-0.009	-0.019
		(0.042)	(0.045)
MD/PhD		0.076	0.016
		(0.039)	(0.046)
PhD		0.060	0.049
		(0.039)	(0.041)
Biomedical science degree		0.041	0.073
		(0.088)	(0.101)
Physical science degree		0.052	0.094
		(0.084)	(0.089)
Social science degree		0.017	0.031
		(0.087)	(0.097)
Prior T32 Predoc Award		-0.024	-0.045
		(0.026)	(0.028)
Prior T32 Postdoc Award		-0.048	-0.033
		(0.031)	(0.033)
Prior NRSA Predoc Award			
Observations		14,268	14,268

*Notes*: Robust standard errors in parentheses. All specifications include controls for IC and council rounds.

*** p<0.001

**p<0.01

*p < .0.05.

*Source*: Authors’ calculations. IMPACII and NIH/NSF Survey of Earned Doctorates.

Recall the analytical sample deletes observations that have peer review scores above the 60th percentile or those that have applied to smaller institutes. As Table 2 indicated, the treatment and control groups in the analysis sample are more closely related than in the full sample (results not shown). The joint hypothesis that the institute and council round fixed effects were significantly different from zero is tested and the hypothesis is rejected (p < .000) in both cases. Thus, the argument that applicants are relatively homogeneous is supported by the analysis, conditional on the review score, council round, and institute. The probit models in Table 3 are the basis of the propensity score estimates used in our matching models in S1 Appendix.

#### Nearest-neighbor matching

Given sensitivity of the propensity score matching (PSM) to the propensity score specification (see S1 Appendix for details) and recommendations to use alternative methods [42], nearest-neighbor matching is used as our preferred method. Throughout, the Mahalanobis distance for nearest-neighbor matching is used. Let *X*^*T*^ be the set of control variables for those receiving the fellowship award and *X*^*C*^ be the set of control variable for non-awardees. The Mahalanobis distance is given by:

$$D=(X^{T}-X^{C})\prime V^{-1}(X^{T}-X^{C})$$

and *V* is the covariance matrix of *X*. Mahalanobis distance will reduce differences between the treated and control groups by an equal percentage for each covariate in the matrix *X*.

The authors experiment with a variety of specifications of the covariate list and matching approach for the nearest neighbor matching. First, they match on the review score only since it is a significant determinant of fellowship funding, and it could not be included in the PSM approach. Second, they match on the review score, institute and council round. Third, they match on review score and council round and impose exact matching on institute. Fourth, they match on all of the variables in the second column of Table 3 and impose exact matching on the institute. About 1,000 observations are dropped that do not have an exact match, leaving 13,653 in the analysis sample. This analysis concentrates on the estimates of the ATE in the last column of Table 4 where the number of covariates is the largest. The nearest neighbor matches increase the number of research program grant awards by 0.10, the number of research program grant applications by 0.73, the probability of a research program grant award by 6.3 ppt, the probability of an elite independent research award by 4.6 ppt, and reduces the probability of never applying for subsequent funding by 9.8 ppt. These estimates are somewhat smaller than the PSM estimates in S1 Appendix but still statistically significant.

**Table 4:** Nearest neighbor full sample and CEM sample ATE estimates (differ by specification of matching covariates).

	(1)	(2)	(3)	(4)
VARIABLES				
Number RPG Awards	0.025	0.072*	0.050	0.101**
	(0.028)	(0.036)	(0.037)	(0.031)
Number RPG Applications	0.428***	0.483***	0.366*	0.726***
	(0.102)	(0.135)	(0.146)	(0.108)
Probability RPG Award	0.040***	0.044***	0.041***	0.063***
	(0.009)	(0.012)	(0.012)	(0.010)
Probability R01 Award	0.026**	0.034**	0.031**	0.046***
	(0.008)	(0.011)	(0.011)	(0.009)
Probability of Never Applying	-0.077***	-0.079***	-0.073***	-0.098***
	(0.010)	(0.014)	(0.014)	(0.011)
Observations	13,653	13,653	13,653	13,653
*Coarsened Exact Matching—Nearest Neighbor Estimates*				
Number RPG Awards	-0.045	0.044	0.045	0.116***
	(0.034)	(0.037)	(0.037)	(0.030)
Number RPG Applications	0.156	0.378**	0.378**	0.777***
	(0.125)	(0.144)	(0.143)	(0.115)
Probability RPG Award	0.015	0.039**	0.039	0.063***
	(0.011)	(0.013)	(0.013)	(0.011)
Probability R01 Award	0.010	0.032***	0.033**	0.051***
	(0.010)	(0.012)	(0.012)	(0.010)
Probability of Never Applying	-0.046***	-0.070***	-0.070***	-0.092***
	(0.014)	(0.016)	(0.016)	(0.012)
Observations	8,630	8,630	8,630	8,630

*Note*: Robust standard errors in parentheses.

*** p<0.001

**p<0.01

*p < .0.05.

*Source*: Authors’ calculations, IMPACII and NIH/NSF Survey of Earned Doctorates, 1996 to 2008.

Coarsened exact matching (CEM) for sample selection can improve the balance of the matching estimator by exactly matching on categorical values of the data [43]. The coarsened data have significantly improved balance and balances nonlinearities and interactions in the sample. This analysis begins by using the CEM algorithm to perform exact matches by institute and fiscal year of funding. Next, the authors use nearest-neighbor matching and same covariates to estimate the ATE, and these estimates inherit the balance properties from the CEM procedure. These estimates appear in the bottom of Table 4. Despite the sample size reduction from 13,653 to 8,630, the nearest-neighbor matches after the CEM algorithm are remarkably similar to the results from the top panel in Table 4.

### Discussion

Overall, these findings fit within and expand the already existing literature on the relationship between training and future career outcomes [3–5, 7, 16]. While others have come to similar conclusions, our analysis updates the impact of funding on more recent cohorts and corrects for a common error in methodology. We provide evidence that an elite fellowship application process and award matter for future engagement as an independent researcher. While this analysis does not test what components of the postdoctoral fellowship might drive the overall findings of impact on future career outcomes, one can identify some of the major differences between fellowship program and other training grants. The NRSA postdoctoral fellowship, for example, requires young scientists to independently develop, under the supervision of a mentor, a research proposal for grant submission early in their career. Possibly, the personalized interaction with mentors in explaining, justifying, and exploring research ideas stemming from the independent thinking of the trainee could help early career scientists *lean in*.

The act of taking the lead and ownership in writing the grant proposal application can also be a valuable learning experience that reduces hurdles and the intimidation of writing a complex grant application in future years. Furthermore, receiving the award allows researchers to start down the path of becoming independent researchers earlier than their colleagues working as postdocs on an already established principal investigator’s project. Early career scientists appointed to a training grant, for example, do not have to develop a research grant proposal since the principal investigator on the project is the faculty mentor who has already written the grant and simply invites postdoctoral researchers (through their own method of interview and selection process) to work in their lab. While these individuals might also be independent thinkers and lead the development of their own research ideas in the mentor’s lab, it is not necessarily a requirement for employment.

No matter the particular mechanism driving outcomes, elite fellowships for postdoctoral scientists appear to increase the probability that awardees will receive research funding later in their career. In an era where major federal agencies are focused on improving the scientific enterprise for early career scientists, these results provide strong evidence to the science policy community and leaders that using mechanisms like the NRSA F32 fellowship to prepare and sustain a biomedical research workforce does achieve the goal of keeping scientists in federally funded research careers. These results are also consistent with the recommendations of NIH’s Biomedical Workforce Working Group [44] and the National Academies of Sciences, Engineering, and Medicine’s Next Generation Researchers Initiative [45], which both suggest that the number of postdoctoral students receiving fellowships should increase.

A limitation of this study is that it does not allow one to examine the extent to which funding from other agencies or research organizations affects those that do and do not receive NRSA F32 funding. However, if individuals were able to receive support from other sources in early career and then develop their federally funded research, we would expect this to bias our estimates downwards. Thus, our results may be considered a lower-bound on the impact of fellowships on subsequent funding and an independent research career.

In Fiscal Year 2018, one NRSA F32 award cost the federal government no more than $60,000 per individual. If a fellowship lasts, on average, two years, then the total cost of funding postdoctoral fellowship training via this mechanism is around $120,000 per individual. Our study shows that making that kind of investment today in young scientists increases the chance that they lean in in the future and remain in a scientific research career.

Congress used to require the National Research Council to conduct a periodic review of the impact of training programs [13]. Furthermore, several researchers and policymakers have noted an increasing length of time spent in training as graduate students or postdocs [46, 47]. These reports have called for policies designed to promote researcher independence. One viable option would be to expand the number of independent elite fellowship awards available.

## Conclusions

During the time of this study, the total number of elite postdoctoral fellowship awards granted decreased; however, their impact as an early career training mechanism to keep individuals in federally funded, future, independent science careers was robust. Of interest to note, the number of fellowship applications decreased during the years when federal funding of biomedical research doubled. Applicants most likely shifted from applying for their own research grants to being signed on to their mentor or supervisor’s grant as the decrease in applicants occurred when funding for postdoctoral researchers on elite independent research grants and research program grants became more available. Postdoctoral appointments on these types of grants did not require postdocs to develop a research proposal distinct from their mentors’ research in order to be appointed. This may influence the direction of their careers and should be studied further.

This study is related to work where similar fellowship awards were modeled as a regression discontinuity and the peer review scoring mechanism was used as a cut off to determine the causal effect of funding at the margin [16]. We found that these methods have significant limitations. In fact, our results underscore the importance of testing the validity of the RDD approach before applying the methods. Federal agencies take their job seriously with staff devoting time, energy, and effort to selecting candidates who have the right mix of institutional support, innovative ideas, and alignment with institute scientific priorities. Candidates are not solely selected based on the peer review score. This means, however, that at least for these elite fellowships, regression discontinuity is not an optimal method for this analysis.

Given the homogeneous nature of F32 applicants, we used matching methods to identify the effect of NRSA funding on outcomes. We found through close examination of the data that the traditional methods using RDD to study the impact of funding was inappropriate. Future work studying the causal effects of science funding will need to grapple with this issue and, before RDD methods are used, researchers will need to test for the validity of RDD assumptions just as researchers currently do with other causal research designs such as difference-in-differences methods.

This paper finds robust results that overall elite fellowships keep postdoctoral researchers in federally funded science at higher rates than those who did not receive the award. Along the margin of being funded or not, the postdoctoral fellowship award mechanism significantly improves the probability of receiving subsequent NIH funding and launching an independent research career. The average treatment effect of a postdoctoral award (for all applicants) increases the future probability of receiving federally funded research awards by anywhere from 4.0 to 6.3 percentage points and the probability of receiving an elite independent research award by anywhere from 2.6 to 4.6 percentage points on average.

Overall, this study provides evidence demonstrating the positive impact of an elite fellowship program on retaining scientists in the scientific research workforce and informs policymakers about the value of future investments in the program. These findings demonstrate that targeted, focused training programs associated with independent research can have a significant impact on keeping individuals in academic science and engaged in federally funded science. Elite fellowship programs can be seen as a viable mechanism for this purpose. Future research should address the issues of cost-benefit and whether the amount of investment in these trainings programs matches the return.

Identifying concrete actions to advance the frontier of science is essential. How, when, and under what mechanisms we train the next generation of scientists matters. This study provides limited yet robust evidence that the priorities of federal agencies to elicit the development of research proposals from early career scientists genuinely improves their future ability to stay attached to the innovative federally funded research pipeline.

## Supporting information

S1 Appendix Propensity score matching results [41, 42, 48]. (DOCX)
