# Supplementary material for: Advancing biomedical science through investments in elite training
Source: PLoS One. 2023 Feb 2;18(2):e0272230. doi: 10.1371/journal.pone.0272230 (PMC9894467; doi:10.1371/journal.pone.0272230)
Supplement: S1 Appendix — (DOCX) [file pone.0272230.s001.docx]

**Appendix A. Propensity score matching results**

Table A1 presents the propensity score matching (PSM) estimates of the ATE and ATT for the analysis sample. As mentioned previously, if the peer review score is included in the propensity score estimates, the propensity score becomes too precise and the matching algorithm breaks down [41, 42]. Thus, the propensity score estimates include institute and council round fixed effects and the covariates listed in column 3 of Table 3. The first row of Table A1 shows the ATE of the fellowship award on the number of research program grant awards, number of research program grant applications, the probability of a research program grant, the probability of an elite independent research award and the probability of never applying for additional funding. The ATE estimates indicate that the fellowship award increases the number of research program grant awards by 0.15; increases the number of research program grant applications by 0.73; increases the probability of a research program grant award by 8.2 ppt; increases the probability of an elite independent research award by 6.0 ppt; and decreases the likelihood of never applying for subsequent funding by 11.2 ppt. The ATT estimates are remarkably similar in size and magnitude.

Table A1. Propensity Score Match Estimates by Outcome

|  | Number of | Number of |  |  | Probability |
| --- | --- | --- | --- | --- | --- |
| VARIABLES | RPG awards | RPG applications | Probability RPG | Probability R01 | never  RPG |
| ATE |  |  |  |  |  |
| Full Sample | 0.149*** | 0.731*** | 0.082*** | 0.060*** | -0.112*** |
|  | (0.026) | (0.098) | (0.009) | (0.008) | (0.010) |
| Trimmed .1,.9 | 0.127*** | 0.584*** | 0.064*** | 0.048*** | -0.098*** |
|  | (0.026) | (0.109) | (0.009) | (0.008) | (0.011) |
| Trimmed .33, .67 | 0.111*** | 0.671*** | 0.067*** | 0.048*** | -0.102*** |
|  | (0.030) | (0.129) | (0.011) | (0.010) | (0.013) |
| ATT |  |  |  |  |  |
| Full Sample | 0.159*** | 0.775*** | 0.088*** | 0.065*** | -0.118*** |
|  | (0.030) | (0.113) | (0.010) | (0.009) | (0.012) |
| Trimmed .1,.9 | 0.143*** | 0.605*** | 0.068*** | 0.050*** | -0.100*** |
|  | (0.031) | (0.134) | (0.011) | (0.010) | (0.013) |
| Trimmed .33, .67 | 0.117** | 0.642*** | 0.070*** | 0.049*** | -0.108*** |
|  | (0.036) | (0.160) | (0.012) | (0.011) | (0.015) |
| Observations | 14,273 | 14,273 | 14,273 | 14,273 | 14,273 |
| Full sample = 14, 273 observations | | |  |  |  |
| Trimmed 1 = 14,021 | |  |  |  |  |
| Trimmed 2 = 6,516 | |  |  |  |  |

*Note*: Robust standard errors in parentheses. *** p<0.001, **p<0.01, *p<.0.05.

*Source*: Authors’ calculations. IMPACII and NIH/NSF Survey of Earned Doctorates, 1996 to 2008.

In order for the estimates to be considered valid, there should be considerable overlap in the propensity score estimates of the awardees and non-awardees. S1 Fig Panel A shows the propensity score overlap for the analysis sample. The mass of the kernel density estimate of the propensity score for receiving an award lies to the left of that for not receiving an award. Trimming the tails of the propensity score distribution below 0.1 and above 0.9 improves the overlap [41]. The second row of Table A1 presents the ATE estimates after trimming. The estimated effects of the fellowship award on the ATE and ATT fall somewhat after trimming but remain statistically significant. S1 Fig Panel B indicates that the overlap in propensity scores appears quite similar. Finally, trimming the propensity score optimizes the common support [48]. This analysis trimmed the propensity scores below 0.33 and above 0.67. S1 Fig Panel C indicates that the overlap has improved considerably after this trimming. The estimated effect of the ATE and ATT remain significant but are smaller. The ATE estimates after the 0.33 and 0.67 trimming indicate that the fellowship award increases the number of research program grant awards by 0.11, increases the number of research program grant applications by 0.67, increases the probability of a research program grant award by 6.7 ppt, increases the probability of an elite independent research award by 4.8 ppt, and decreases the likelihood of never applying for subsequent funding by 10.2 ppt. As before, the ATT estimates are remarkably similar in size and magnitude.

Although one cannot test the unconfoundedness assumption directly, one researcher [41] recommends using PSM on pseudo-outcomes that occur prior to the award treatment. Given the SED data, the authors evaluate whether the fellowship award predicts the probability that an applicant has a PhD degree, the applicant’s field of highest degree is in biomedicine, and the applicant’s doctoral funding was from a fellowship or scholarship. Table A2 presents these results and finds no significant impact of the fellowship award on these pseudo-outcomes.

Table A2. Counterfactual Treatment Effects With Pseudo Treatments

|  | (1) | (3) | (5) |
| --- | --- | --- | --- |
| VARIABLES | PhD degree | Biomedical  degree | Fellowship or scholarship PhD funding |
|  |  |  |  |
| ATE | 0.007 | 0.001 | 0.005 |
|  | (0.007) | (0.010) | (0.010) |
|  |  |  |  |
| ATT | 0.005 | 0.007 | 0.004 |
|  | (0.008) | (0.012) | (0.012) |
|  |  |  |  |
| Observations | 14,273 | 14,273 | 14,273 |

*Note*: Robust standard errors in parentheses.

*Source*: Authors’ calculations, IMPACII and NIH/NSF Survey of Earned Doctorates, 1996 to 2008.

PANEL A PANEL B

PANEL C PANEL D

S1 Fig. Score Overlap by Award

*Notes:* Panel A is the full analysis sample. Panel B is the analysis sample with score > .1 and score < .9. Panel C is the analysis sample with score > .33 and score < .67. Panel D is the CEM sample.

*Source:* Authors’ calculations. National Institutes of Health IMPACII administrative records, 1996-2008.
